# Mining and exploration of rehabilitation nursing targets for colorectal cancer

**DOI:** 10.18632/aging.205739

**Published:** 2024-04-16

**Authors:** Ruipu Li, Jie He, Zhijie Ni, Jie Zhang, Xiaoqian Chi, Chunbo Kang, Zhongbo Li, Xubin Li

**Affiliations:** 1 Gastrointestinal Rehabilitation Center, Beijing Rehabilitation Hospital Affiliated to Capital Medical University, Shijingshan 100144, Beijing, China; 2 Department of Colorectal Surgery, China Aerospace Science and Industry Corporation 731 Hospital, Fengtai, Beijing, China

**Keywords:** DSCC1, GINS1, colorectal cancer, bioinformatics, differentially expressed genes

## Abstract

Background: There are often subtle early symptoms of colorectal cancer, a common malignancy of the intestinal tract. However, it is not yet clear how MYC and NCAPG2 are involved in colorectal cancer.

Method: We obtained colorectal cancer datasets GSE32323 and GSE113513 from the Gene Expression Omnibus (GEO). After downloading, we identified differentially expressed genes (DEGs) and performed Weighted Gene Co-expression Network Analysis (WGCNA). We then undertook functional enrichment assay, gene set enrichment assay (GSEA) and immune infiltration assay. Protein-protein interaction (PPI) network construction and analysis were undertaken. Survival analysis and Comparative Toxicogenomics Database (CTD) analysis were conducted. A gene expression heat map was generated. We used TargetScan to identify miRNAs that are regulators of DEGs.

Results: 1117 DEGs were identified. Their predominant enrichment in activities like the cellular phase of the cell cycle, in cell proliferation, in nuclear and cytoplasmic localisation and in binding to protein-containing complexes was revealed by Gene Ontology (GO). When the enrichment data from GSE32323 and GSE113513 colon cancer datasets were merged, the primary enriched DEGs were linked to the cell cycle, protein complex, cell cycle control, calcium signalling and P53 signalling pathways. In particular, MYC, MAD2L1, CENPF, UBE2C, NUF2 and NCAPG2 were identified as highly expressed in colorectal cancer samples. Comparative Toxicogenomics Database (CTD) demonstrated that the core genes were implicated in the following processes: colorectal neoplasia, tumour cell transformation, inflammation and necrosis.

Conclusions: High MYC and NCAPG2 expression has been observed in colorectal cancer, and increased MYC and NCAPG2 expression correlates with worse prognosis.

## INTRODUCTION

Colorectal cancer (CRC) is a neoplasm that starts in the colon or large bowel. It characteristically develops in the cells of the mucous membrane lining these gastrointestinal structures [1]. The risk of developing bowel cancer increases with age and mainly affects people aged 50 and over. People aged 60 and over account for the majority of cases. The incidence of bowel cancer differs around the world, with more common in developed countries and less common in developing countries [2]. Historically, bowel cancer has been more common in men than in women. However, in recent years, the incidence of bowel cancer in women has been on an upward trend, and this gender difference has been gradually decreasing [3]. Colorectal cancer cells have the ability to grow aggressively, infiltrate adjacent tissues and organs, and, in more advanced stages, travel through the bloodstream and lymphatic system to distant sites, forming distant metastases [4]. Colorectal cancer is a formidable challenge, both in terms of mortality and complexity of treatment. It is a disease that manifests itself primarily through symptoms such as irregular bowel movements, rectal bleeding (haemorrhoids) and the potential development of anaemia. This underscores the need for nuanced and comprehensive medical interventions. Typical treatments involve a range of approaches, including surgical removal of tumours, radiotherapy, chemotherapy, targeted therapy and other methods [5]. Completely eradicating cancer cells, stopping their spread and preserving the patient's quality of life as much as possible is the ultimate goal of treatment. With its potential to affect an individual's psychological well-being, colorectal cancer can lead to challenges such as anxiety, depression and other emotional difficulties, which can reduce overall quality of life and create economic burdens [6]. Bowel cancer is a complex interaction of genetic changes, environmental factors and lifestyle choices. A key role is played by genetic mutations that promote uncontrolled cell proliferation and inhibit apoptosis. Chronic inflammation can induce DNA damage and cellular mutations in the intestinal tract. Obesity, with its associated chronic low-grade inflammation, has effects on insulin sensitivity, hormonal pathways and cellular signalling. Diets high in fat and low in fibre, including red and processed meats, contribute to cellular damage and inflammation. At the same time, habits such as smoking and excessive alcohol consumption expose the bowel to toxic substances that can damage the bowel lining and may put you at risk of bowel cancer [7]. It is of paramount importance to delve more deeply into the molecular intricacies of colorectal cancer.

It is at the intersection of computer science, maths, statistics and biology, and brings them together in harmony. Their job is to painstakingly process and analyse biological data to unravel the complex web of organism structure, function and evolution [8]. Bioinformatics is a powerful tool for tackling biological challenges that have proved elusive to traditional experimental methods. It excels at unravelling the intricacies of complex gene regulatory networks and probing the molecular basis of disease. Beyond simply analysing genomic and proteomic data, bioinformatics has evolved to comprehensively explore established and novel gene products. These technologies are at the cutting edge of proteomic research, enabling a more sophisticated understanding of different aspects of the proteome, contributing to a more holistic view of biological systems [9].

However, the association among MYC, NCAPG2 and colorectal cancer (CRC) is still unclear. This survey therefore seeks to harness the power of computational biology to reveal the key genes that differentiate bowel cancer from normal tissue. Through a meticulous exploration involving enrichment analysis and pathway analysis, this research aims to shed light on the intricate roles played by MYC and NCAPG2 in the development of colorectal cancer. These findings are being validated using public data sets, which will add depth and credibility to MYC and NCAPG2's important implications in colorectal cancer.

## METHODS

### Download the colorectal cancer data sets

Colorectal cancer data sets GSE32323 and GSE113513 were extracted respectively from GEO database, specifically generated from GPL6102 and GPL14951. GSE32323 consisted of 17 bowel cancer cases and 17 normal cases, while GSE113513 consisted of 14 bowel cancer cases and 14 normal cases. The primary objective was to investigate differentially expressed genes (DEGs) in relation to colorectal cancer.

### To batch processing

An orchestrated approach was used for the integration and disentangling of the multiple data sets. Initially, the R software package facilitated the amalgamation of the GSE32323 and GSE113513 datasets. Using the R package In SilicoMerging, a consolidated matrix was obtained by merging these data sets. Batch effects were then removed with the help of the remove patch effect routine in limma R (version 3.42.2). The resulting batch-independent matrix was used as the basis for subsequent analysis.

### Screening of DEGs

The fusion matrices of GSE32323 and GSE113513 were subjected to a fusion clustering and alignment using the R package limma. The Benjamini-Hochberg approach was employed to refit *p*-values to increase statistical robustness. False discovery rate (FDR) was applied to calculate fold change (FC) using a stringent set of criteria with a threshold of *P* < 0.05 and FC >1.5. The identification of differentially expressed genes (DEGs) was facilitated by generating a visually informative volcano map.

### Weighted gene co-expression network analysis (WGCNA)

First, a batch correction process was performed to use the genome description matrix by merging GSE32323 and GSE113513. Then, excluding the upper 50% of the smallest genes, the Median Absolute Deviation (MAD) was calculated for each gene. Outlying genes and outlying samples were identified and excluded using the goodSamplesGenes method from the R package WGCNA. A coexpression scaled network was then assembled using WGCNA. For further refinement of the module analysis, the genetic differences within the modules have been calculated and cut lines have been selected on the module tree. In particular, modules with a merging distance of less than 0.25 were consolidated. Importantly, the grey module was considered unassignable to a gene module collection.

### Functional enrichment analysis

The goal of the study was to identify functional differences within the gene lists through extensive computational analysis using GO and KEGG methods. By submitting the gene list to the following KEGG Web API (https://www.kegg.jp/kegg/rest/keggapi.html), the current KEGG Annotation for this gene was retrieved. Using this information as a background, genes were assigned and subjected to clustering analysis using the R package clusterProfiler (version 3.14.3). Gene set enrichment results were considered statistically significant if they met the criteria of a lowest gene set of 5, a highest gene set of 5000, and a *P*-value < 0.05 with a FDR <0.25.

In addition to this, the Metascape database, which is a valuable resource for the comprehensive annotation and analysis of genes, was also used. The gene enrichment analysis results were further explored and visualised using the Metascape database (http://metascape.org/gp/index.html). This process provided a thorough understanding of the functional implications of the identified gene differences and allowed the export of relevant gene lists for more detailed study.

### GSEA

Gene Set Enrichment Analysis (GSEA) was purchased from the official GSEA homepage. Two different groups of colorectal cancer and normal tissue samples were stratified. Subsets were then downloaded from the Molecular Signatures Database, which allowed the evaluation of the corresponding pathways and underlying mechanistic pathways. Using the genetic expression profile and group of phenotype, GSEA was performed with a lowest gene set size of 5 and a highest gene set size of 5000, using 1000 resampling iterations. Results were considered statistically significant if they met the criteria of a *P*-value of < 0.05 and FDR of < 0.25. In addition, GSEA was extended to include Gene Ontology (GO) and Kyoto Encyclopedia of Genes and Genomes (KEGG) annotations to provide a comprehensive exploration of the colorectal cancer genetic landscape.

### Immunoinfiltration analysis

The computational tool CIBERSORT (http://CIBERSORT.stanford.edu/) was used as a commonly accepted technique to assess infiltration of immune-system cells in colorectal cancer. An integrated bioinformatics approach using the CIBERSORT software package was adopted to comprehensively analyse the combined gene expression matrix of the colorectal cancer datasets GSE32323 and GSE113513. Using the principles of linear regression, this method facilitated the unfolding of the immune cell component expressivity matrices and provided estimates of immune cell abundance. A confidence level (*P* < 0.05) was used to ensure that data were included with sufficient reliability and accuracy.

### Construction and analysis of protein-protein interaction (PPI) networks

The Search Tool for the Retrieval of Interacting Genes (STRING, http://string-db.org/) database is a comprehensive platform for aggregating, evaluating and integrating publicly available data on protein-protein interactions, complemented with computational predictions. In this investigation, the index of differentially expressed genes was entered into the STRING database to establish a PPI network to predict key genes (confidence >0.4). Cytoscape software, renowned for its capabilities in biological network analysis and two-dimensional (2D) visualisation, was used to visualise and predict these key genes within the PPI networks.

The process involved importing the PPI mesh into Cytoscape and identifying the clusters with the strongest correlations using the MCODE algorithm. Five different tools (MCC, MNC, EPC, Eccentricity, Betweenness) were applied to predict the most robustly correlated genes and their intersections. A list of core genes contributing to a broader understanding of the underlying molecular interactions was identified through visualisation.

### Survival analysis

When examining clinical survival data for colorectal cancer from The Cancer Genome Atlas (TCGA), we employed the R software package maxstat (version: 0.7–25) to determine the ideal RiskScore truncation value associated with core genes. By defining a lowest sample size of 25% and a highest sample size of 75%, we derived the ideal truncation values. Patients were then stratified into a hyper and hypo group based on these values. The surpass function of the R software package survival was then employed to examine the prognographic differences between these groups, and the difference was assessed for significance using the test for significance (log-rank test).

In order to assess the impact of each of the independent core genes on the prognosis of colorectal cancer, we observed any notable changes in effect. Receiver operating characteristic (ROC) analyses were also performed by means of the R package pROC (version 1.17.0.1). Patient follow-up at specific time points (365, 1095 and 1825 days) was included in the ROC analyses. The pROC ROC function and ci function assessed area under the curve (AUC) alongside confidence intervals, providing a comprehensive assessment of core gene prognostic score diagnostic value.

### Heat map of gene expression

Visual representations in the form of heat maps illustrating the level of regulation of the core genes clustered within the PPI network were generated using the R package heatmap. This approach allowed us to visually explore the nuanced differences in expressing colorectal cancer and regular tissue samples within the GSE32323 and GSE113513 datasets.

### CTD analysis

The Comparative Toxicogenomics Database (CTD) is an invaluable resource that seamlessly integrates a wide range of data, including chemical, genetic and functional information. This comprehensive database facilitates the exploration of interactions between phenotypes and disease-related environmental exposures, providing considerable convenience for investigating potential disease and drug mechanisms.

For further analysis, the identified ‘key genes’ were entered into the CTD website and the primary diseases associated with these ‘key genes’ were identified. The complex relationships between each gene and their respective diseases were further clarified by generating a differential expression radar map for each gene using Excel. This process has added a layer of visual clarity to the complex interplay between the core genes and their associated diseases and has improved our understanding of the potential mechanisms and relationships involved.

### The miRNA

In our investigation, we utilised the capabilities of TargetScan (http://www.targetscan.org). This online database is highly regarded for its ability to predict and analyse miRNAs and their target genes. Our use of TargetScan focused on the meticulous screening of miRNAs that are intimately involved in the regulation of key differentially expressed genes (DEGs). The goal was to reveal the complex regulatory relationships between miRNAs and the key genes we identified.

### Data availability

The datasets generated during and/or analyzed during the current study are available from the corresponding author on reasonable request.

## RESULTS

### Exploration of genes with altered expression

**Figure 1:**
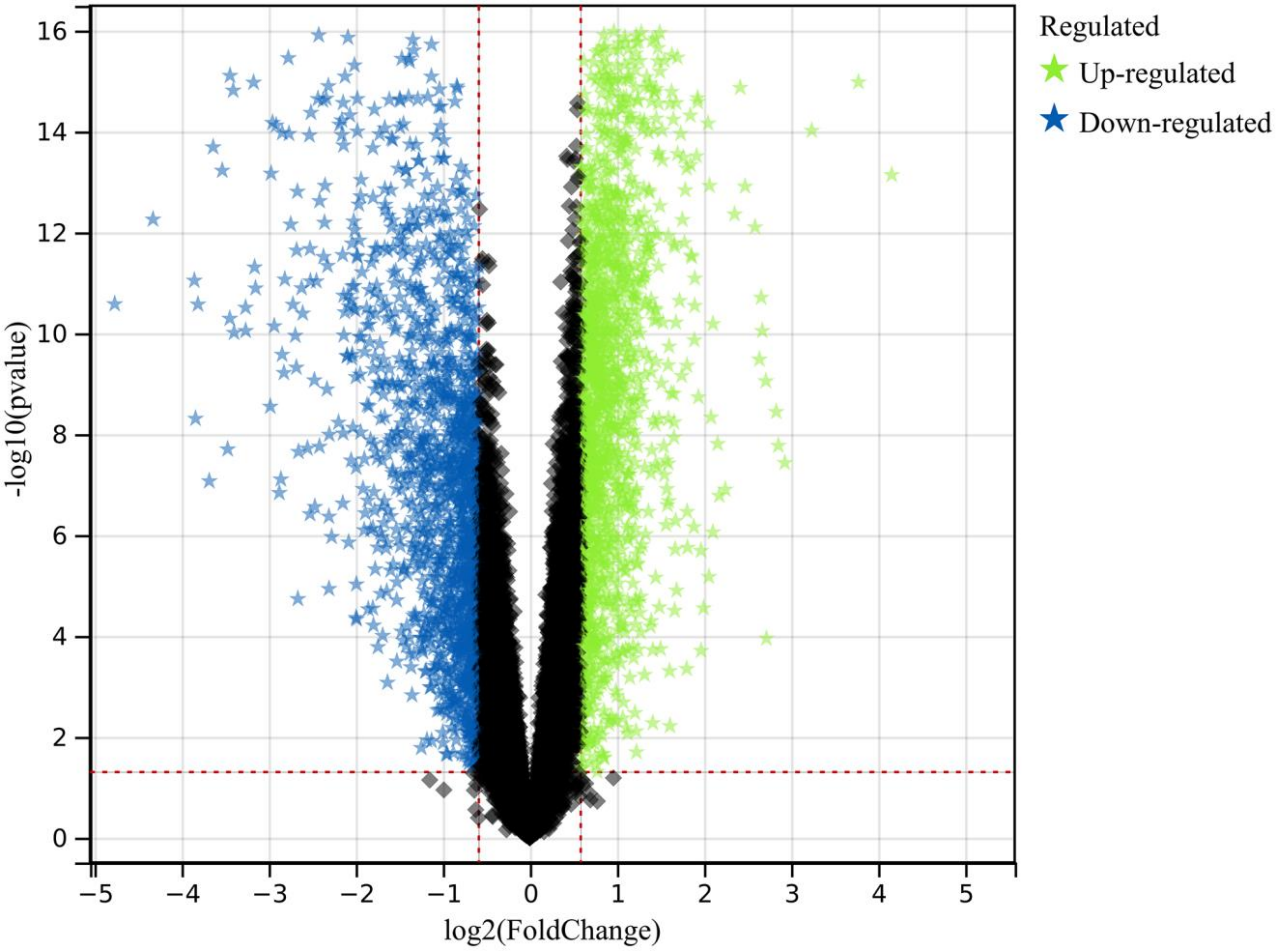
**Exploration of differentially expressed genes (DEGs).** A comprehensive set of 1117 DEGs was identified and analyzed, shedding light on the molecular distinctions underlying colorectal cancer.

In this investigation, we used pre-defined cut-off criteria to identify genes with altered expression levels within the merged dataset of the colorectal cancer datasets GSE32323 and GSE113513. As shown in Figure 1, the result of this analysis was a comprehensive set of 1117 DEGs.

### Functional enrichment analysis

#### DEGs

**Figure 2:**
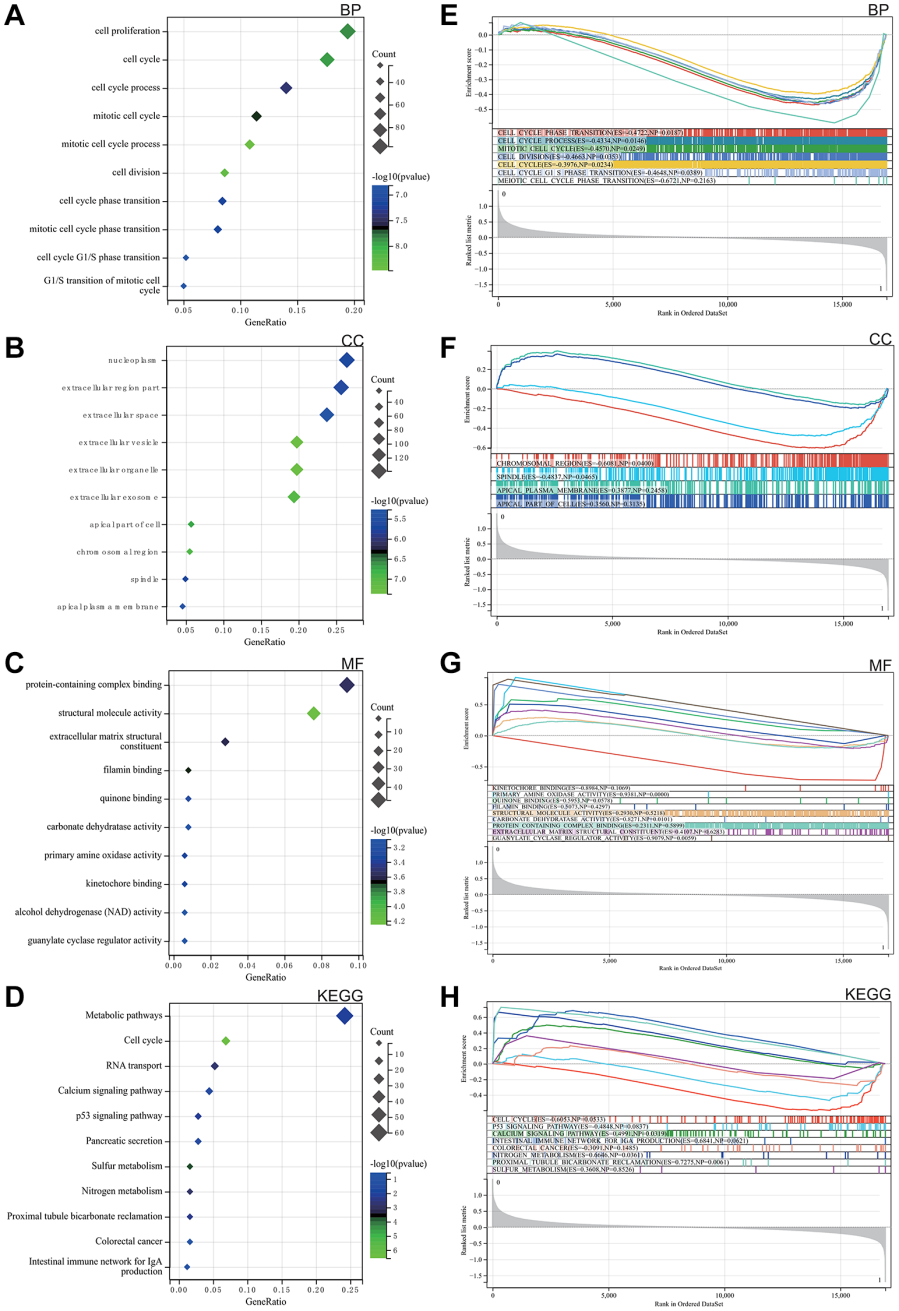
**Analysis of the functional enrichments.** (**A**–**C**) GO. (**D**) Analysis of the KEGG. (**E**–**H**) GSEA.

As elegantly illustrated in Figure 2A–2C, based on Gene Ontology (GO) analysis, the enriched terms predominantly revolved around key biological functions including cell cycle, cell reproduction and nuclear content containing protein complexes.

The KEGG analysis highlighted significant pathways, with particular emphasis on calcium signalling, P53 signalling, colorectal cancer and the intestinal immune network of IgA, as shown in Figure 2D.

#### GSEA

GSEA was performed to explore potential enrichment terms for genes that were not differentiated, confirming and extending the finding from the differentiated genes. The combined matrix of colorectal cancer datasets GSE32323 and GSE113513 underwent GSEA, revealing major enrichments in DEGs related to mitotic cell cycle, protein complexes, broader cell cycle, as well as calcium and P53 signalling pathways. These revealing results are visually presented in Figure 2E–2H.

### Metascape enrichment analysis

**Figure 3:**
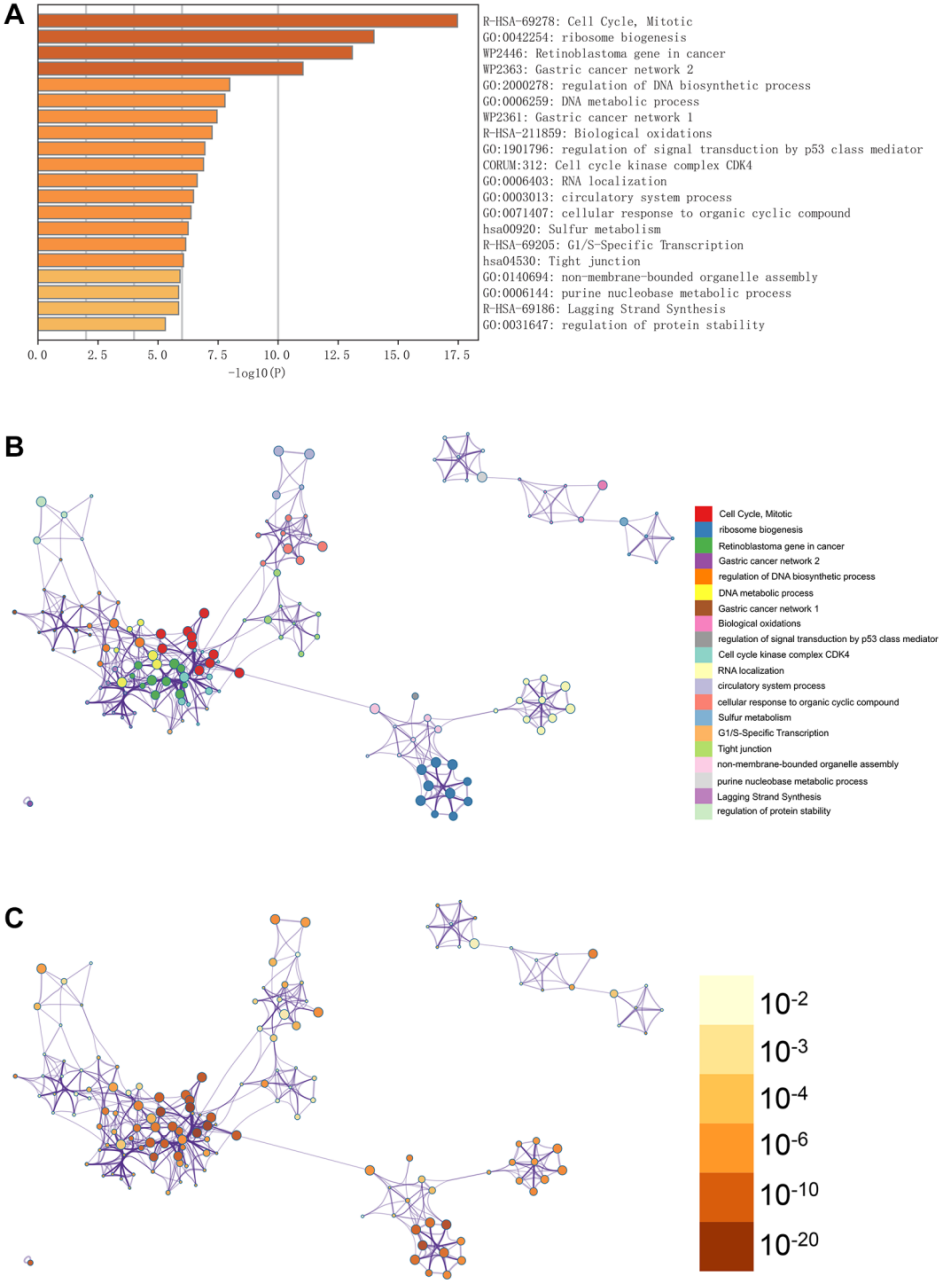
**Enrichment analysis in the metascape.** (**A**) In the metascape enrichment project, the GO enrichment project is visible. (**B**) The enrichment network coloured by enrichment terms. (**C**) The enrichment network coloured by *p*-values.

**Figure 4:**
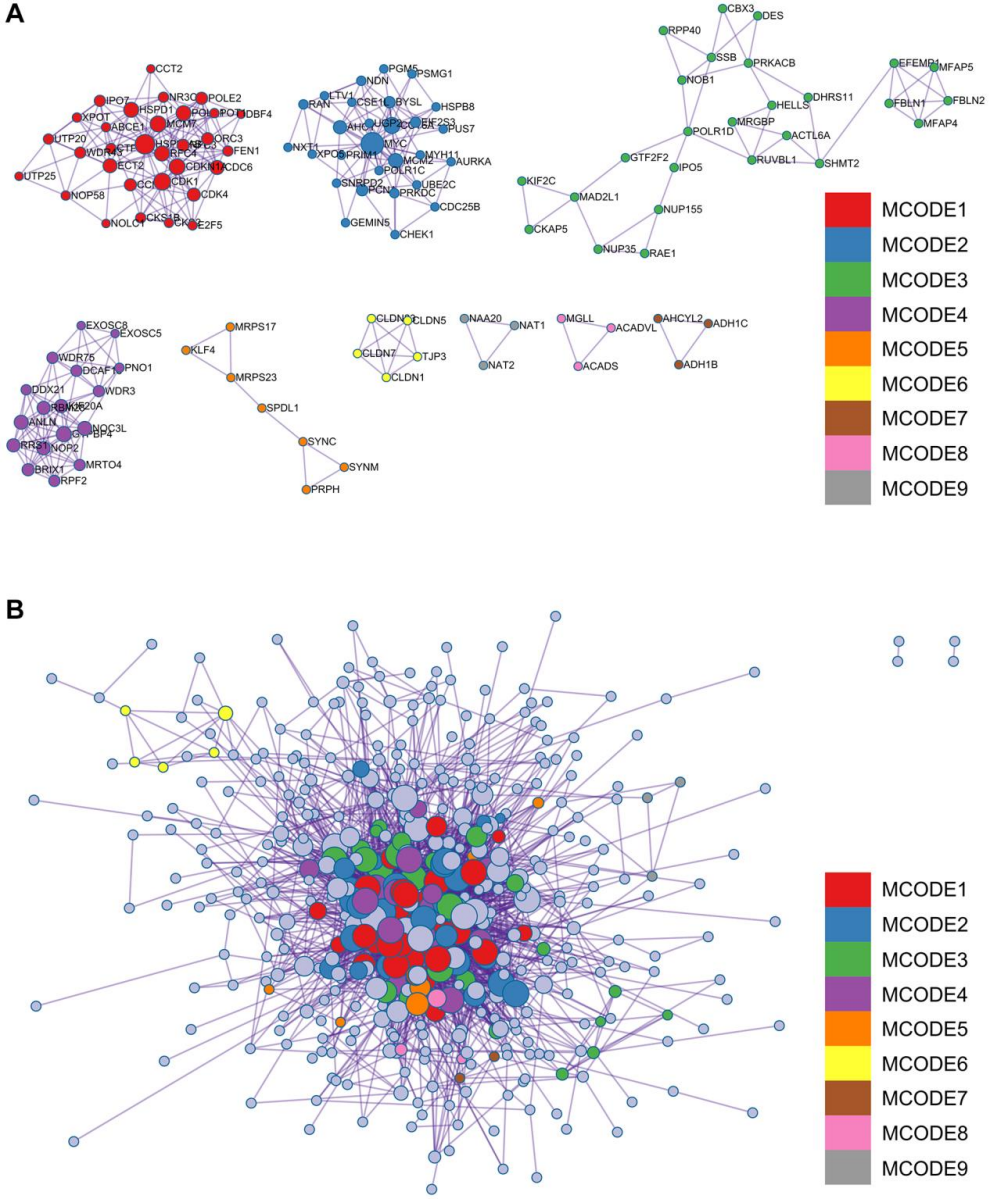
**Analysis of the metascape enrichment.** (**A**) The different modules of MCODE. (**B**) PPI network in the MCODE.

In Figure 3A, the GO enhancement project unfolds graphically in the Metascape enrichment exploration. Simultaneously, we generated an output visualising the enrichment network, where the colouring reflects the enrichment terms and their corresponding *p*-values (Figures 3B, 3C and 4). This visual representation artfully conveys the interconnectedness and confidence of each enrichment term, enhancing clarity and depth of understanding.

### Immunoinfiltration analysis

**Figure 5:**
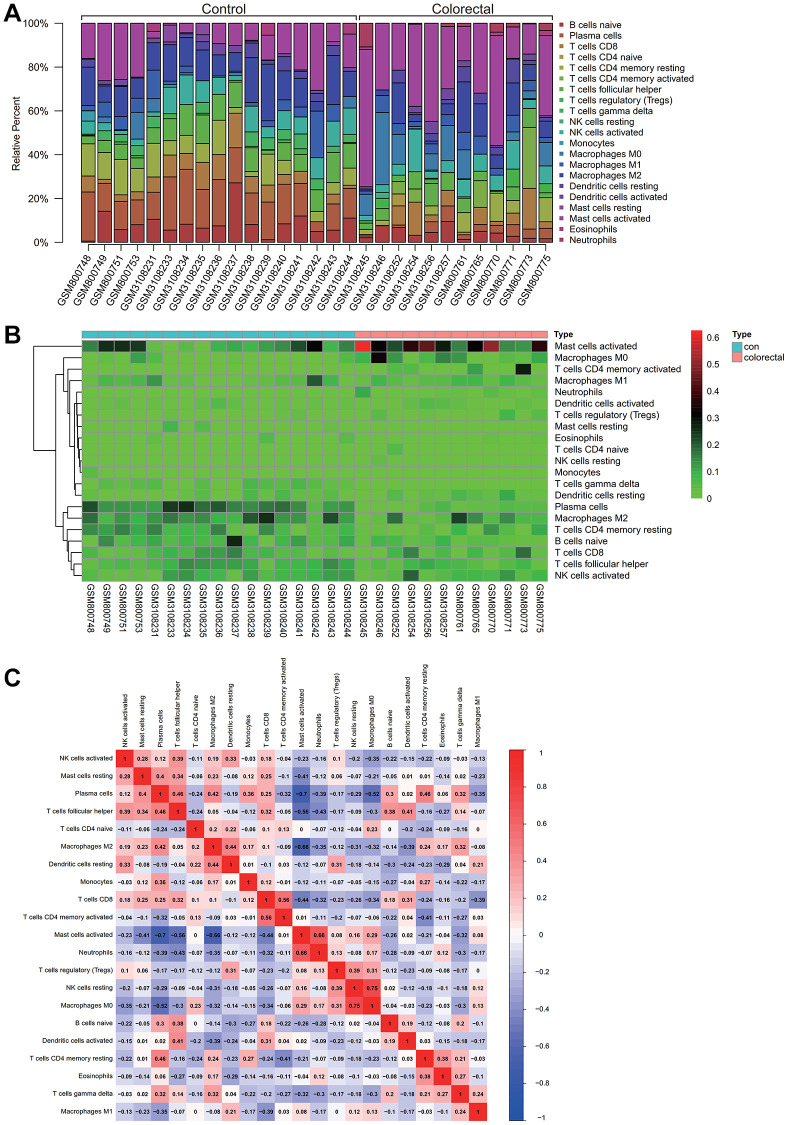
**Analysis of immune infiltration.** (**A**) Activated M2 macrophages and mast cells comprised a high proportion of the sample. (**B**) A Heatmap of immune cell expression in the dataset. (**C**) The correlative analysis was conducted on immune cell infiltration, the common expression patterns between components of immune cells, macrophages M2 high expression, activated mast cells can express is low.

Insights into immune cell composition were obtained by comprehensive analysis of the combined matrix from the colorectal cancer datasets GSE32323 and GSE113513 by means of the CIBERSORT software package. The proportions of immunological cells within the total genomic pattern were determined at a 95% confidence level. In particular, M2 and activated mast cells were found to be dominant components in these samples (Figure 5A).

In a hematogram depicting the expression levels of immune cells in the dataset, a particularly increased representation of activated mast cells was observed in colorectal cancer samples (Figure 5B). To unravel potential relationships between immune cell infiltrations, correlation analysis was performed, which revealed common expression patterns. Notably, an inverse correlation was observed between high M2 macrophage expression and low activated mast cell expression. As shown in Figure 5C, this complex interplay may have implications for colorectal cancer progression.

### WGCNA

**Figure 6:**
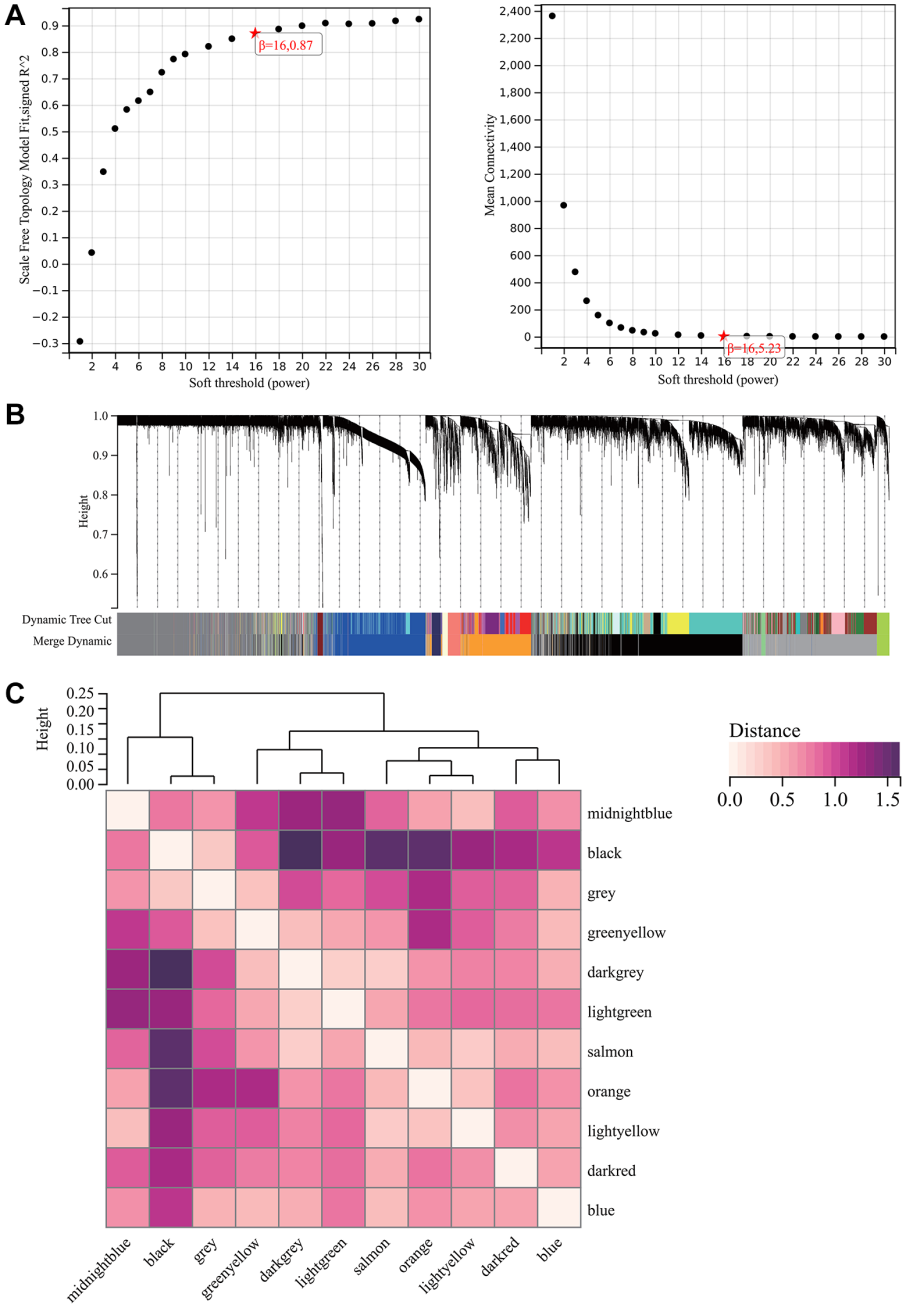
**WGCNA.** (**A**) β = 16, 0.87. β = 16, 5.23. (**B**) All the genes involved in the production of the 11 modules. (**C**) The interaction between the most important module.

**Figure 7:**
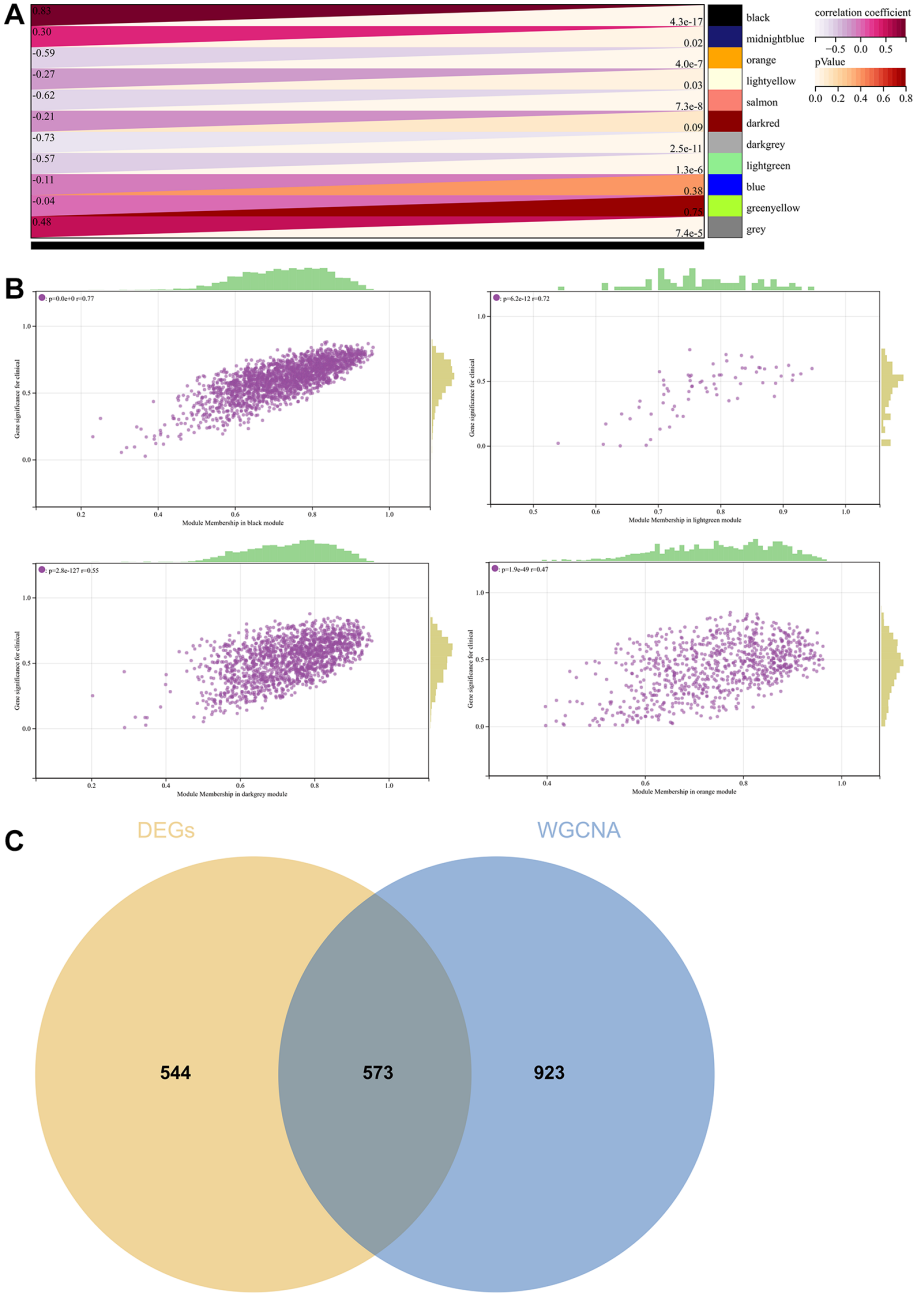
**WGCNA.** (**A**) A module and a phenotypic correlation heatmap. (**B**) The corresponding hub gene of GS and MM correlation scatter plot. (**C**) Venn diagram of WGCNA and the differentially expressed genes screened by DEGs, and their intersections.

A critical step in WGCNA is the soft threshold power selection. Using network structure analysis, we set the performance soft-threshold to 9, which is the bottom performance that achieves a scale-freeness topological fit index of 0.9 (Figure 6A). A clustered hierarchical tree was then established, revealing 11 distinct assemblies of genes (Figure 6B). In summary, in addition to generating a correlation heatmap between modules and phenotypes (Figure 7A), in-depth analysis focused on interactions within an essential module (highlighted in Figure 6C). A scatterplot (Figure 7B) was also generated to explore the correlation between gene significance (GS) and module membership (MM).

We performed correlations between module signature vectors and gene abundance, emphasising a strict cutting standard (|MM| >0.8), to identify critical hub genes. Ten strongly associated genes in clinically meaningful modules were clustered as hub genes. To further elucidate the molecular landscape, a Venn diagram was constructed to illustrate the overlap between genes identified by WGCNA and those identified as DEGs (Figure 7C).

### Protein - protein interaction (PPI) network building and analysis

**Figure 8:**
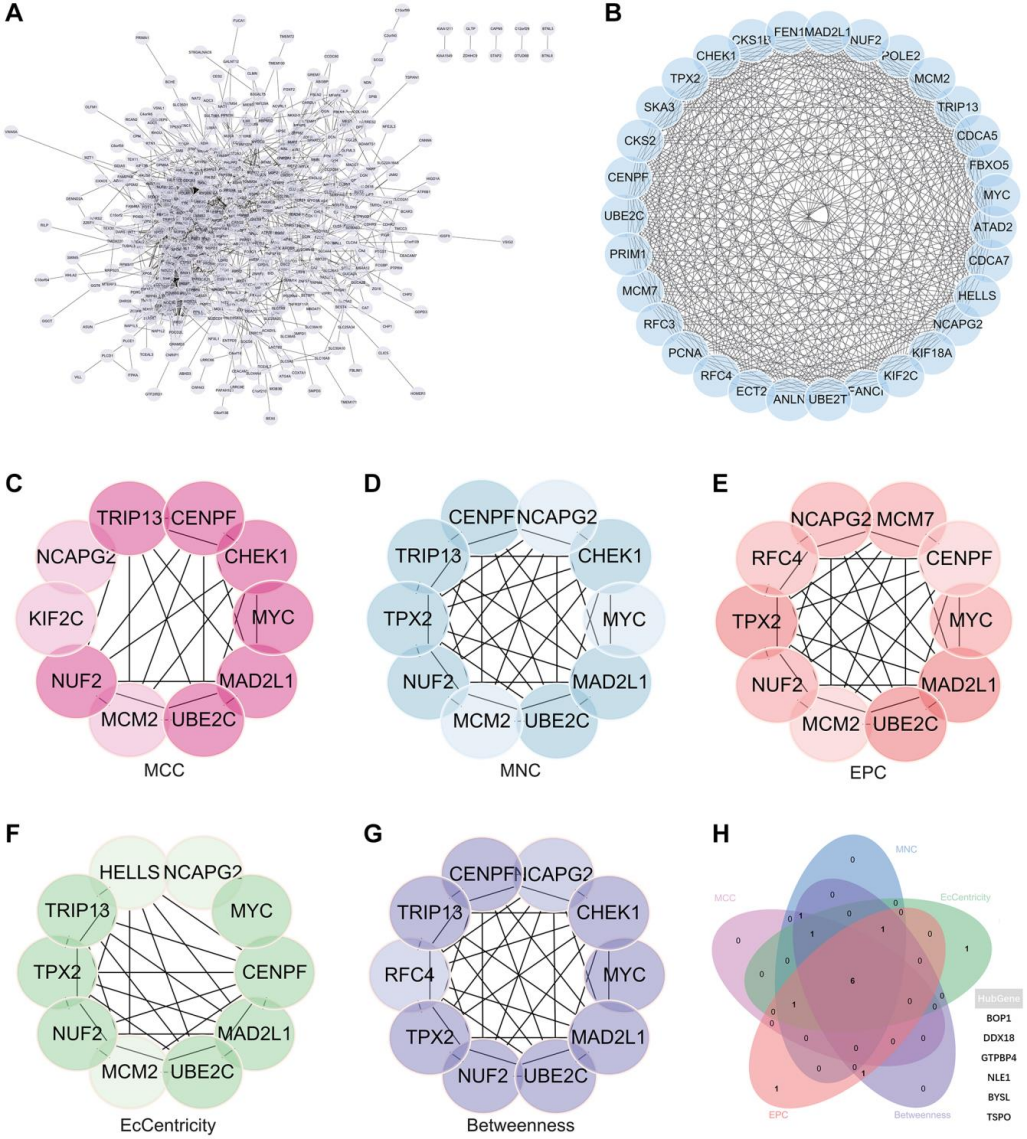
**Protein - protein interaction (PPI) network building and analysis.** (**A**) PPI net. (**B**) The nuclear cluster. (**C**–**G**) Five algorithms (MCC, MNC, EPC, EcCentricity, Betweenness) were used for the identification of the core genes. (**H**) To obtain the combination as core genes, the Venn diagram was made.

A complex PPI network was established by integrating WGCNA with gene lists from DEGs. This network was meticulously generated with the STRING online database and analysed using Cytoscape software (Figure 8A), yielded a recognisable core set of genes as highlighted in Figure 8B.

In an effort to identify central genes within this network, we applied five different algorithms (MCC, MNC, EPC, EcCentricity, Betweenness), which resulted in the identification of potential central genes, each of which is shown in Figure 8C–8G. An elaborate Venn diagram analysis merging the results of these algorithms accurately pinpointed the core genes, as depicted in Figure 8H. At the end of this analysis, six genes (MYC, MAD2L1, CENPF, UBE2C, NUF2 and NCAPG2) emerged as the focal points of interest within the complex molecular landscape.

### Survival analysis

**Figure 9:**
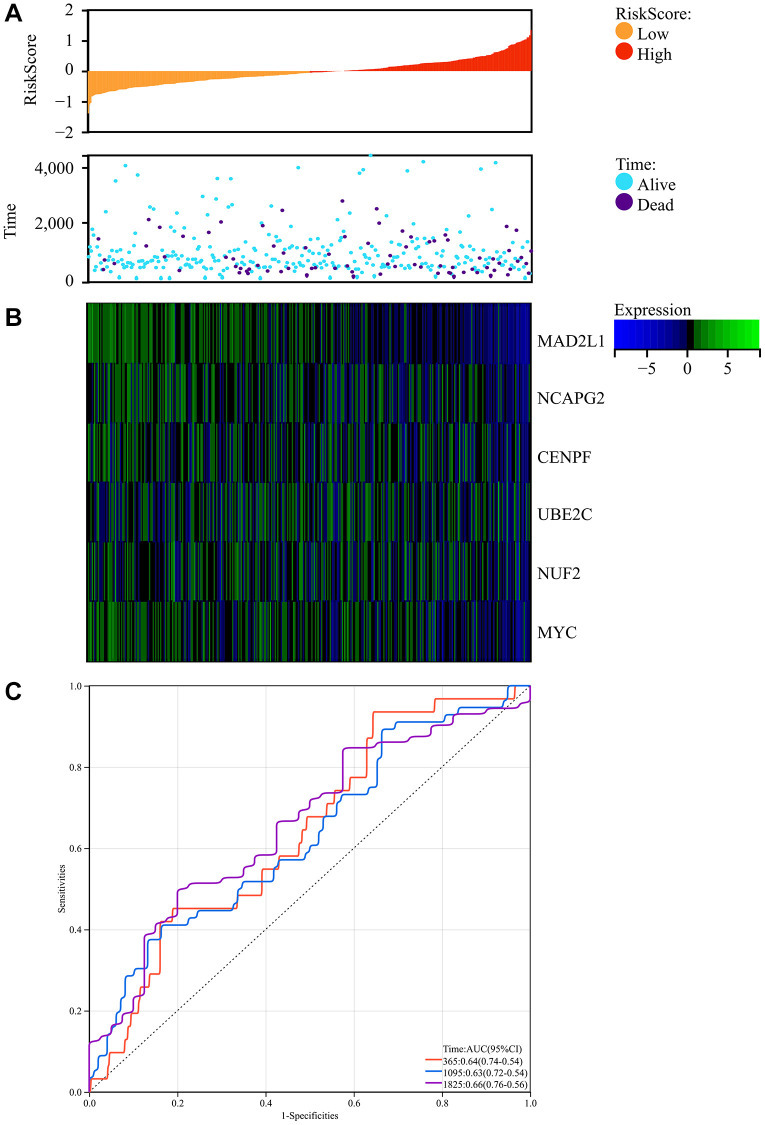
**Survival analysis.** (**A**) Prognosis score relationship map and expression difference heat map of core genes between colorectal cancer and normal tissue specimen. (**B**) The core genes were protective factors, showing decreasing expression trends with increasing risk score. (**C**) Risk score ROC curve.

We obtained colorectal cancer survival data from TCGA and explored the complex association of colorectal cancer risk scores with gene expression patterns. The research produced a comprehensive map showing the relationship between prognostic scores and differences in the methylation of key genes between colorectal cancer and regular test tissue samples. Notably, there was a significant decrease in patient survival as the risk score increased, with the low-risk group having significantly longer periods and rates of survival than the high-risk group (Figure 9A).

In addition, the identified core genes functioned as protective factors, showing a consistent downregulation as risk score increased (Figure 9B). To estimate the predictive ability of the risk scores, we constructed an ROC curve, which showed promising results with AUC values indicating the forecasting potential of the risk scores (Figure 9C).

**Figure 10:**
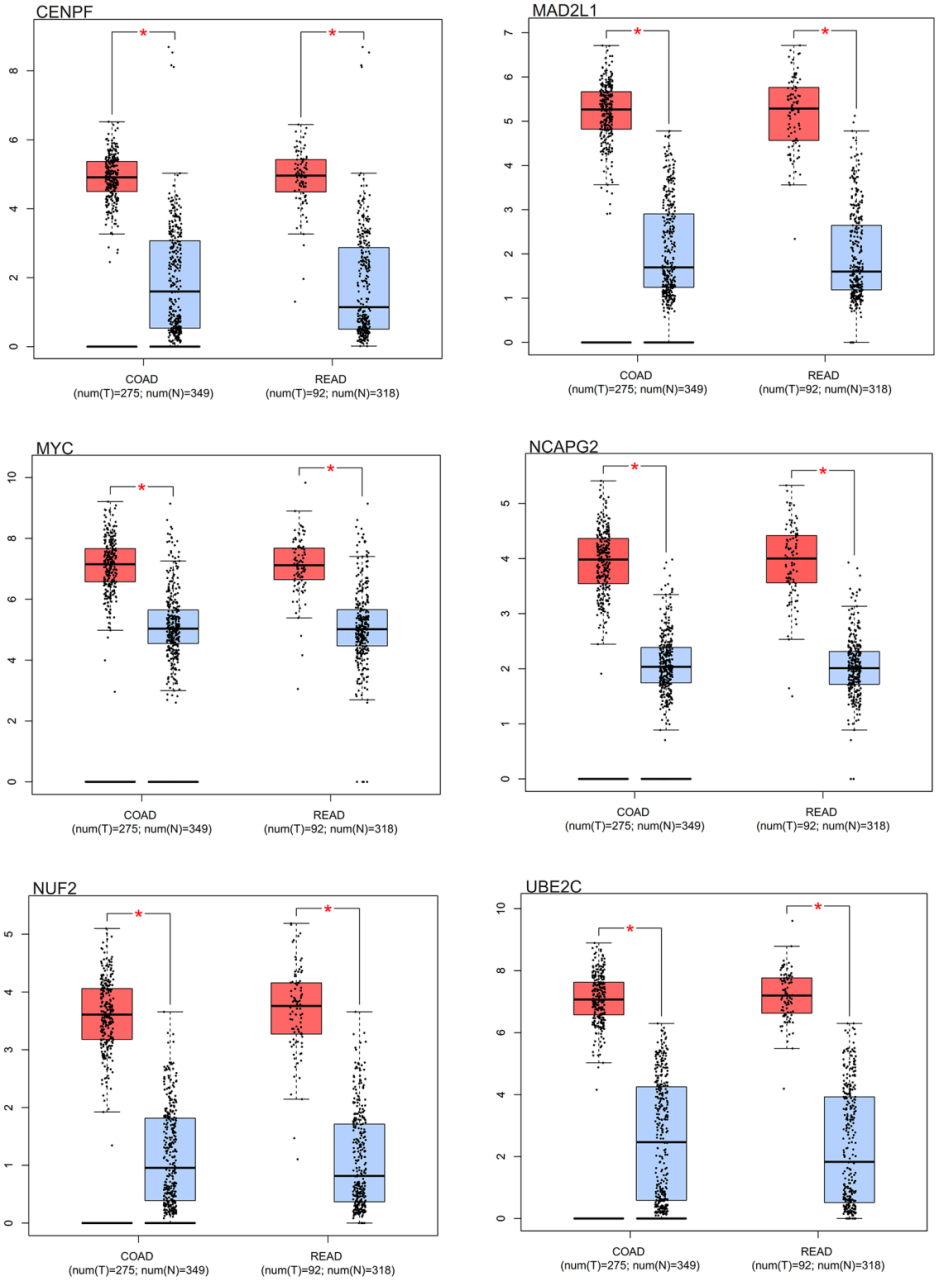
**Survival analysis.** Box results of colorectal cancer core genes.

Distinct expression patterns were observed in the box plot analysis of the core genes in colorectal cancer. Notably, the core genes (MYC, MAD2L1, CENPF, UBE2C, NUF2, NCAPG2) had differential expression in colorectal cancer samples in comparison to normal samples. As elegantly illustrated in Figure 10, these core genes showed an increased expression in colorectal cancer samples, contrasting with a decreased expression in normal samples.

### Heat map of core gene expression

**Figure 11:**
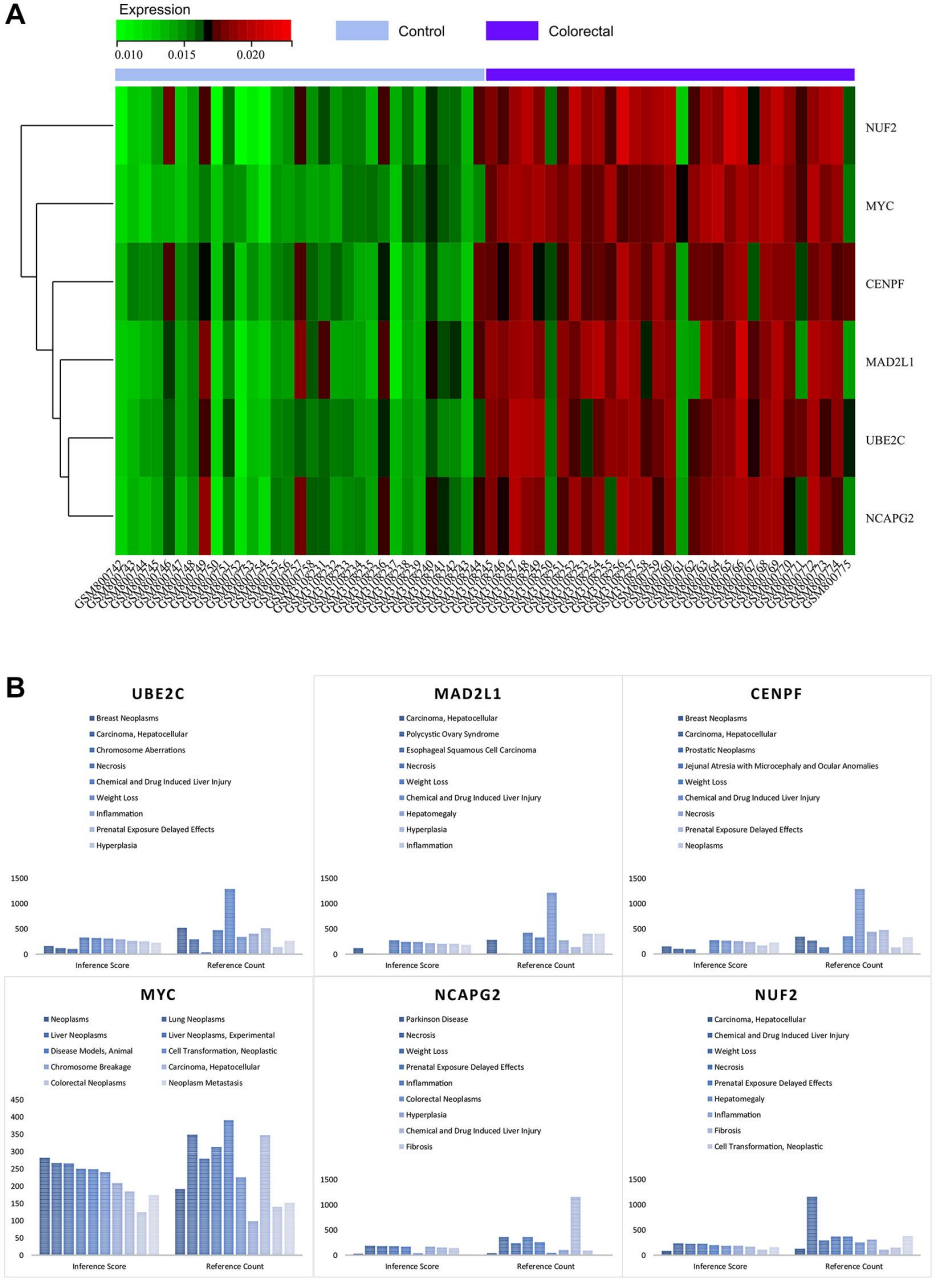
(**A**) Heat map of the expression of core genes. (**B**) Analysis of the CTDs.

By visualising the expression levels in the combined matrix of GSE32323 and GSE113513, a striking pattern was revealed. The core genes, namely MYC, MAD2L1, CENPF, UBE2C, NUF2 and NCAPG2, showed elevated expression in colorectal cancer samples, in stark contrast to their comparatively lower expression in normal samples (shown in Figure 11A). This intriguing observation leads to speculation about the potential regulatory role of these master genes in the context of colorectal cancer.

### CTD analysis

As part of this research, we submitted the cluster gene list to the CTD website, which provided valuable insights into the diseases closely associated with the cluster genes. Specifically, the core genes (MYC, MAD2L1, CENPF, UBE2C, NUF2, NCAPG2) showed associations to colorectal neoplasia, tumour cell transformation, inflammation and necrosis, as elegantly illustrated in Figure 11B. This nuanced exploration has deepened our understanding of the intricate interplay between these core genes and a variety of disease processes.

### Hub gene associated miRNA prediction and functional annotation

Here, the index of target genes was incorporated into TargetScan, which revealed relevant miRNAs to improve the understanding of gene expression regulation (Table 1). Interestingly, MAD2L1 was found to be associated with miRNAs such as hsa-miR-6088, hsa-miR-143-3p and hsa-miR-4770. CENPF genes showed associations with miRNAs such as hsa-miR-302c-3p and hsa-miR-520f-3p. In addition, the NUF2 gene was associated with hsa-miR-599. The intricate regulatory networks involving these genes and their associated miRNAs are revealed by this nuanced investigation.

**Table 1:** A summary of miRNAs that regulate hub genes.

	**Gene**	**MIRNA**		
**1**	**MAD2L1**	hsa-miR-6088	hsa-miR-143-3p	hsa-miR-4770
**2**	**CENPF**	hsa-miR-302c-3p.2	hsa-miR-520f-3p	
**3**	**NUF2**	hsa-miR-599		
**4**	**MYC**	None		
**5**	**UBE2C**	None		
**6**	**NCAPG2**	None		

## DISCUSSION

Colorectal cancer (CRC) stands out as a widespread and formidable malignancy that makes a significant contribution to morbidity and mortality around the world. Its aggressive nature is underscored by its tendency to become invasive and metastasize [10]. A thorough understanding of the molecular intricacies of colorectal cancer is of paramount importance in the further development of targeted drug discovery. The elevated expression of MYC and NCAPG2 in colorectal cancer is one of the key findings of this study. In particular, they highlight the importance of these molecular markers in predicting disease outcome, as higher levels of MYC and NCAPG2 correlate with poorer prognosis. MYC encodes a pivotal protein serving as a transcription factor with the capacity to intricately influence the expression of numerous genes [11]. One of the commonly expressed tumour genes in human cancer is MYC. The proto-oncogene MYC plays a critical role in cell cycle control, cell growth and differentiation under normal physiological conditions. Its functions include orchestrating processes such as proliferation and metabolism [12]. The maintenance of a balance in the expression of MYC is essential for the normal function of the cell. Perturbations in this delicate balance, particularly by overexpression, have been linked to the development and progression of many cancers [13].

MYC has been shown to affect gene expression throughout the body, in both normal and cancerous cells. Through interactions with multiple factors, this multifaceted protein stimulates different stages of transcription, including initiation, pausing, release and elongation. MYC protein is involved in chromatin resolution, transcript initiation and elongation, and mRNAhalf-life [14]. Degradation of MYC is essential for the release of transcriptional silencing. The pathogenic manifestations of MYC are primarily attributed to aberrant expression levels due to increased transcription, chromosome gain or loss.

MYC influences the tumour microenvironment to facilitate cancer evasion of host immune surveillance and is a central player in orchestrating cancer growth and immune evasion [15]. A key target in cancer therapy, MYC comprises three homologous genes - C-MYC, N-MYC and L-MYC. It is one of the most commonly aberrant genetic drivers in human tumours [16]. MYC orchestrates global gene expression and dictates the complex orchestration of cell proliferation, differentiation, cell cycle progression, metabolism and apoptosis [17]. Simply activating MYC in tissue culture is sufficient to induce many of the characteristics associated with tumour cells. In addition, in the absence of external growth factors, activation of MYC leads to both proliferation and growth of the cells [18]. MYC acts as a universal transcription enhancer that interacts with numerous factors and complexes to control virtually every cellular event. In various malignancies, MYC amplification takes different forms, including chromosomal tandem repeats and extrachromosomal loops. MYC contributes to the formation of complex molecular networks that are critical for controlling cell fate decisions and the intricacies of carcinogenesis through intricate interactions with numerous genes and signalling pathways [19]. Underscoring the multifaceted nature of this regulatory network, upon MYC inactivation, tumour regression is orchestrated by a combination of cell-autonomous processes and non-cell-autonomous mechanisms [20].

MYC is a group of genes that includes c-MYC, N-MYC, L-MYC and others. All of these genes belong to the MYC family. They are involved in processes such as cell growth, differentiation, proliferation and apoptosis [21]. The C-Myc gene has been the subject of much research attention, particularly since the identification of its abnormal activation, which is closely linked to the development and progression of a wide range of tumours. The increased expression of genes such as c-Myc has the potential to drive uncontrolled cell proliferation and to inhibit apoptosis, thereby playing a role in the formation of tumours [22]. The scientific literature documents that c-Myc, as a transactivation factor, triggers the regulation of a large number of genes that are critical for various cellular processes. In particular, c-Myc plays an instrumental role in enhancing the glycolytic metabolism of cancer cells, which has clinical implications in promoting the growth and metastasis of various cancers [23, 24]. The c-Myc protein promotes proliferation by orchestrating the coordination of multiple genes responsible for the cell division cycle and DNA synthesis. This includes upregulating cyclin and nucleotide metabolism enzyme expression, potentially promoting continuous tumour cell division and growth [25]. The over-expression of the c-Myc protein also has the ability to suppress apoptosis, which is a fundamental cellular regulatory mechanism designed to eliminate cells that are damaged or abnormal. Inhibition of apoptosis gives tumour cells the ability to evade programmed cell death. This allows them to continue to grow and metastasise. Research has linked abnormal c-Myc expression to developing colorectal cancer, as well as its effect on glucose metabolism [26]. C-myc has been found to play a role in both the development and evolution of colorectal cancer [27].

Cancer initiation, maintenance and progression have been linked to disruptions in MYC function [28]. As bowel epithelium transforms, MYC is often expressed by various mechanisms that initiate and/or sustain tumor growth [29]. Mounting evidence that MYC plays a central role in tumour initiation, maintenance and progression makes targeting MYC a very attractive strategy for cancer therapy. In many cases of colorectal cancer, there is a considerable increase in the production of the MYC gene, resulting in an overabundance of its protein product (c-MYC). This excessive expression contributes to uncontrolled proliferation of tumor cells, promoting growth and resistance to apoptosis. Therefore, MYC is likely to play an intrinsic role in the progression of colorectal tumours.

NCAPG2 is a protein-coding gene with implications for cell biology and the stability of the genome. As a subunit of the non-SMC condensin II complex, it plays a critical role in chromosome condensation during the cell cycle. By promoting chromosome condensation, it contributes to proper cell division and helps to avoid chromosome instability [30].

NCAPG2 is expressed in different cell types, and the abnormal increase could affect the cell's chromosome assembly and segregation process, thus playing a role in cancer cell proliferation and metastasis [31]. NCAPG2 is a critical constituent of the chromosome lectin II compound. It has a major role in the aggregation and segregation of mitotic chromosomes [32]. NCAPG2 has attracted considerable attention in several cancers as a key player in cell mitosis. Its central roles include critical biological processes such as organelle division, mitosis, segregation of chromosomes, DNA duplication, and control of cell cycle phase transitions and mitotic cleavage [33]. NCAPG2, which is essential for centrosome positioning and cell cycle regulation, is emerging as a potential target for immunoinfiltrative therapies. Many studies have shown that it is upregulated in tumour cells and is involved in tumour proliferation, metastasis and invasion. With respect to glioblastoma, NCAPG2 actively promotes cancer cell progression [34]. NCAPG2 orchestrates the progression of malignant melanoma by fine-tuning both proliferation and metastasis to exert its pro-tumorigenic effects [35]. NCAPG2 emerges as a novel promoter in the setting of metastatic breast cancer, suggesting that it plays an important role in driving breast cancer incipience and progression [36]. NCAPG2 appears promising as a potential biomarker to assess immune response and predict prognosis in pancreatic cancer patients [37]. Enhanced upregulation of NCAPG2 has been shown to correlate with increased inflammatory infiltration and progression of NSCLC [38]. NCAPG2 transcriptional activity promotes liver cancer cell proliferation and migration [39]. NCAPG2 is linked to the immunosuppressive environment of cancer, suggesting an important role in the etiology of colorectal cancer.

MYC and NCAPG2, key players in cancer research, are of paramount importance in unravelling the complexities of colorectal cancer. MYC, when overexpressed, drives unrestrained cell cycle progression and promotes uncontrolled cell proliferation. At the same time, MYC inhibits apoptosis, allowing cancer cells to survive longer and preventing the natural elimination of abnormal cells. On the other hand, NCAPG2, with its potential role in cell cycle and chromosome stability regulation, becomes essential for maintaining the integrality of the genome. Aberrations in MYC and NCAPG2 expression or mutations may serve as valuable biomarkers for colorectal cancer, providing insight into early detection, assessing disease severity and predicting prognosis.

Investigation of MYC and NCAPG2 mutations promises to refine colorectal cancer subtyping, thereby contributing to development of more precise treatment modalities. In addition, both genes are emerging as potential therapeutic targets. This paves the way for interventions designed to disrupt their functions and limit cancer cell survival. A deeper understanding of the complexities of MYC and NCAPG2 mechanisms may elucidate the development of drug resistance and provide opportunities for strategies to overcome resistance challenges and improve therapeutic efficacy.

In spite of the comprehensive bioinformatic analysis, this study has a number of limitations. Experimental validation by gene expression or knockout experiments in animal models is lacking. Therefore, future research efforts should delve deeper into this aspect to provide a more thorough understanding and validation of the findings.

## CONCLUSIONS

Elevated levels of MYC and NCAPG2 in colorectal cancer correlate with poorer prognosis. Based on these findings, MYC and NCAPG2 have been designated as promising molecular targets for the treatment of this disorder. High expression of MYC and NCAPG2 is significantly predictive of clinical outcomes in colorectal cancer patients, indicating their potential clinical relevance. This combination enables a more integrated perspective on the impact of research findings on patient care and treatment. With benefits such as early disease detection, timely intervention and improved overall survival, these findings have significant implications for colorectal cancer patients. This research is also helping to identify the mechanisms underlying the disease and refine therapy strategies, opening up new possibilities for early diagnosis and targeted intervention. Identifying MYC and NCAPG2 as biomarkers may pave the way for personalized treatments tailored to individual biology, optimizing outcomes while minimizing unnecessary side effects.
